# Impact of S-Wave Amplitude in Right Precordial Leads on Improvement in Mitral Regurgitation following Cardiac Resynchronization Therapy

**DOI:** 10.3390/jcdd9050159

**Published:** 2022-05-16

**Authors:** Naoya Kataoka, Teruhiko Imamura, Takahisa Koi, Shuhei Tanaka, Nobuyuki Fukuda, Hiroshi Ueno, Koichiro Kinugawa

**Affiliations:** Second Department of Internal Medicine, University of Toyama, 2630 Sugitani, Toyama 930-0194, Japan; nkataoka@med.u-toyama.ac.jp (N.K.); taka1010@med.u-toyama.ac.jp (T.K.); stanaka@med.u-toyama.ac.jp (S.T.); nhukuda@med.u-toyama.ac.jp (N.F.); hueno@med.u-toyama.ac.jp (H.U.); kinugawa@med.u-toyama.ac.jp (K.K.)

**Keywords:** heart failure, cardiac resynchronization therapy, QRS amplitude

## Abstract

Background: The therapeutic strategy for mitral regurgitation (MR) in patients with advanced heart failure and wide QRS complex who are indicated for both intervention to MR and cardiac resynchronization therapy (CRT), remains unclear. Objective: We aimed to determine electrocardiogram parameters that associate with MR reduction following CRT implantation. Methods: Among the patients with advanced heart failure and functional MR who intended to receive CRT implantation, baseline QRS morphology, electrical axis, PR interval, QRS duration, and averaged S-wave in right precordial leads (V1 to V3) in surface electrocardiogram were measured. The impact of these parameters on MR reduction following CRT implantation, which was defined as a reduction in MR ≥1 grade six months later, was investigated. Results: In 35 patients (median 71 years old, 18 men), 17 (49%) achieved an MR reduction following CRT implantation. Among baseline characteristics, only the higher S-wave amplitude in right precordial leads was an independent predictor of MR reduction (odds ratio 14.00, 95% confidence interval 1.65–119.00, *p* = 0.016) with a cutoff of 1.3 mV calculated through the area under the curve. The cutoff significantly stratified the cumulative incidences of heart failure re-admission and percutaneous mitral valve repair following CRT implantation (*p* = 0.032 and *p* = 0.011, respectively). Conclusions: In patients with advanced heart failure and functional MR, the baseline higher amplitude of S-wave in the right precordial leads might be a good indicator of MR improvement following CRT.

## 1. Introduction

Functional mitral regurgitation (MR) is a serious comorbidity in patients with advanced heart failure [1]. A percutaneous mitral valve repair (PMVR) using MitraClip (Abbott Vascular, Menlo Park, CA, USA) reduces heart failure rehospitalization rate and all-cause mortality in strictly selected patients with functional MR [2]. Alternatively, cardiac resynchronization therapy (CRT) also improves concomitant functional MR in some cases [3]. Given this, a new question arises: which device therapy is suitable for patients with advanced heart failure and functional MR as an initial therapy.

QRS duration is key to considering PMVR or CRT. Among those with advanced heart failure and severe MR, the current guidelines recommend PMVR rather than CRT when the patients had QRS duration ≤120 ms [4,5,6]. CRT is preferred for those with QRS duration >120 ms. However, MR persists or even worsens following CRT implantation in some of them, despite their wide QRS duration [7,8]. Concomitant or early intervention to MR is required for such a cohort. Further optimal patient selection is desired for CRT implantation to enjoy improvement in MR, in addition to the conventional CRT indication.

Recently, we reported a simple electrocardiogram (ECG) predictor of CRT responses in addition to the conventional CRT indication: an S-wave amplitude in right precordial leads [9]. Morphological cardiac reverse remodeling following CRT implantation might be associated with the improvement in MR. Taken together, we hypothesized that baseline high S-wave amplitude in right precordial leads might be a novel predictor of MR improvement following CRT implantation.

## 2. Results

### 2.1. Patient Characteristics 

A total of 35 patients (median 71 years old, 18 men) were included (Table 1). Most of the patients (54%) had mild MR, and nine (26%) patients had severe MR. According to QRS morphology, the number of patients with LBBB was 6 (60%) in MR grade 1, 0 in MR grade 2, 1 (10%) in MR grade 3, and 3 (30%) in MR grade 4; with RBBB was 2 (25%), 2 (25%), 0, and 4 (50%), respectively; with intraventricular conduction disturbance was 5 (100), 0, 0, and 0, respectively; and with right ventricular apical pacing was 6 (50), 2 (17), 2 (17), and 2 (17), respectively, *p* = 0.202. The averaged S-wave amplitude in right precordial leads on median was 1.3 (0.7, 3.0) mV. 

Following CRT implantation, 17 (49%) patients achieved MR reduction. There were no significant differences in the baseline characteristics between those with and without MR reduction, except for left atrial diameter. Regarding ECG parameters, there were significant differences in QRS morphology and S-wave amplitude. Biventricular pacing rate also failed to show the differences between those with and without MR reduction. Of note, the prevalence of LBBB and the amplitude of the averaged S-wave in right precordial leads were higher in the MR reduction group than those without MR reduction. 

As a sub-group analysis, among those without LBBB or RV pacing, the averaged S-wave amplitude was higher in patients with MR reduction (1.6 [1.3–1.8] mV versus 0.5 [0.1–0.8] mV, *p* = 0.029). The trend was similar among those with LBBB or RV pacing (*p* = 0.0356).

### 2.2. Impact of S-Wave Amplitude in Right Precordial Leads on MR Reduction

**Figure 1:**
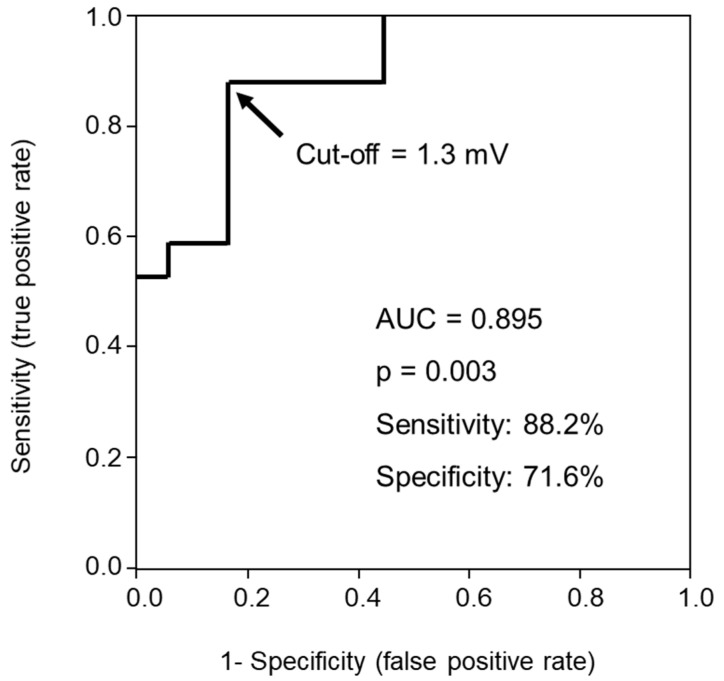
Receiver operating characteristic curve of averaged S-wave amplitude in right precordial leads for predicting mitral regurgitation reduction following cardiac resynchronization therapy.

In the multivariable analyses, the averaged S-wave amplitude in right precordial leads was an independent predictor of MR reduction with an odds ratio 14.00 (95% confidence interval 1.65–119.00, *p* = 0.016) adjusted for left atrial diameter, LBBB, and RBBB (Table 2). ROC analysis showed a cutoff of 1.3 mV for the S-wave amplitude in right precordial leads to best predict MR reduction with an area under the curve of 0.895, a sensitivity of 88.2%, and a specificity of 71.6% (Figure 1). Pre-implant MR severity was not significantly different between those with and without S-wave amplitude in right precordial leads >1.3 mV (11 (61%) in MR grade 1, 1 (6%) in MR grade 2, 2 (11%) in MR grade 3, and 4 (22%) in MR grade 4 versus 8 (47%), 3 (17%), 1 (6%), and 5 (29%), respectively, *p* = 0.595).

**Figure 2:**
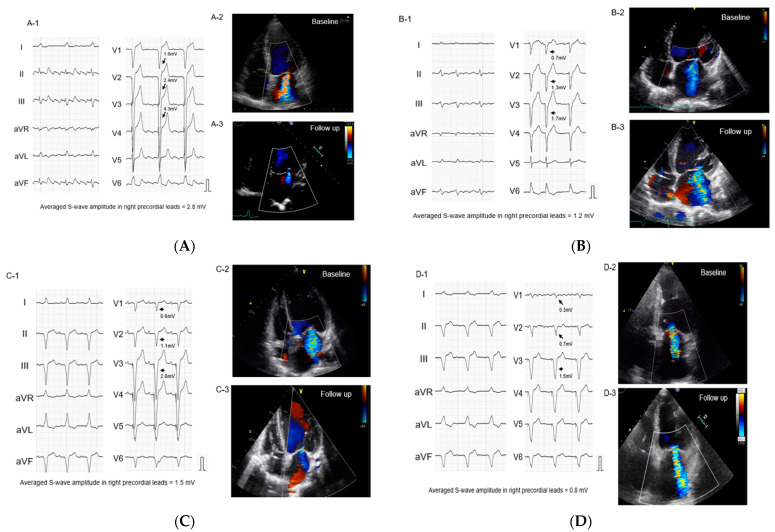
Representative baseline electrocardiograms (left bundle branch block (**A**,**B**), right ventricular apical pacing (**C**,**D**)). Arrows indicate S-waves in right precordial leads. (**A**). Left bundle branch block and averaged S-wave amplitude >1.3 mV: the patient with MR reduction (**B**). Left bundle branch block and the averaged S-wave amplitude <1.3 mV: the patient without MR reduction (**C**). Right ventricular apical pacing and the averaged S-wave amplitude >1.3 mV: the patient with MR reduction (**D**). Right ventricular apical pacing and the averaged S-wave amplitude <1.3 mV: the patient without MR reduction.

Representative ECGs and echocardiograms are displayed in Figure 2. Figure 2A,B present complete LBBBs. The averaged S-wave amplitude in right precordial leads is higher in Figure 2A (2.8 mV), with a considerable improvement in MR, whereas Figure 2B displays a low S-wave amplitude (1.2 mV), with MR remaining the same. Figure 2C,D present right ventricular apical pacing. The averaged S-wave amplitude in right precordial leads is higher (1.5 mV) in Figure 2C, with a considerable reduction in MR, whereas MR persists in Figure 2D with low S-wave amplitude (0.8 mV).

### 2.3. Impact of S-Wave Amplitude in Right Precordial Leads on Other Clinical Outcomes

The reduction rate in the left ventricular end-systolic volume and improvement rate in LVEF 6 months following CRT were not statistically different between those with and without MR improvement (26 ± 36% vs. 9 ± 23%, *p* = 0.113; 27% (17–65%) vs. 27% (8–44%), *p* = 0.313, respectively).

**Figure 3:**
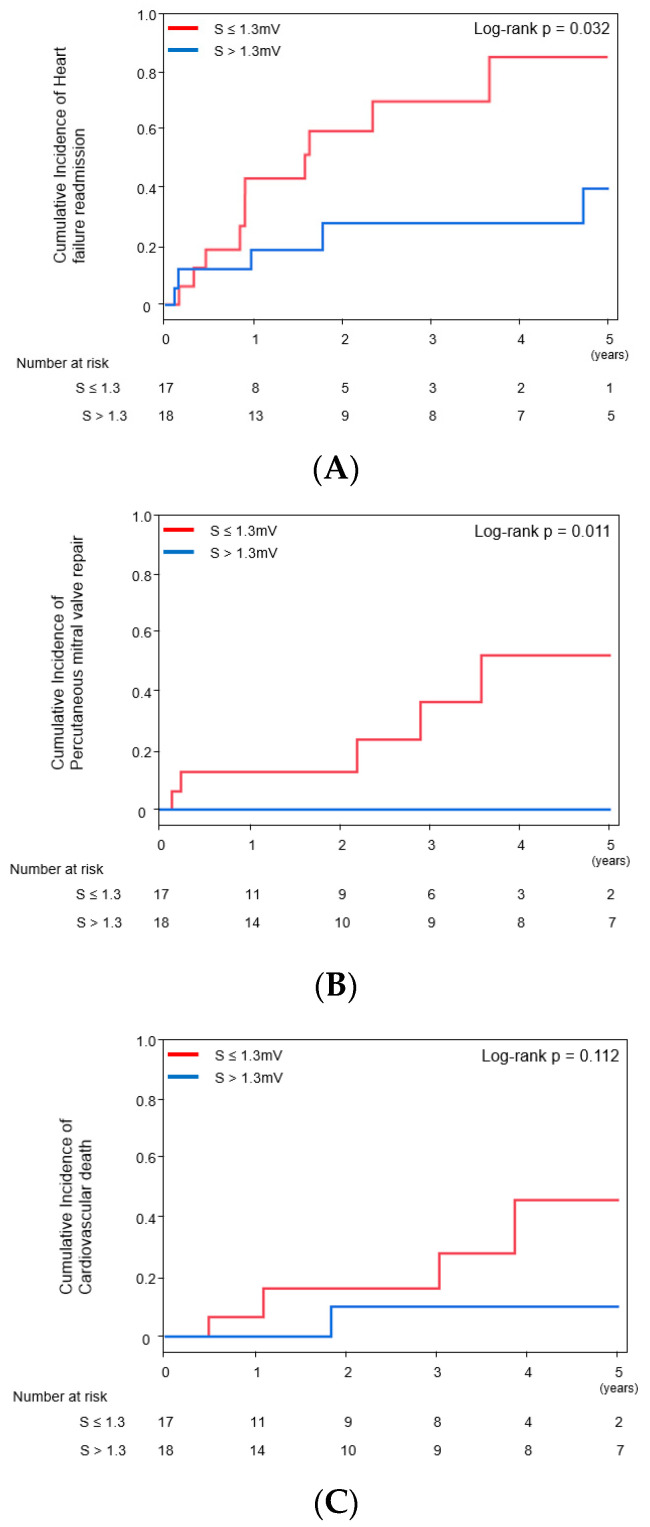
Cumulative incidence of clinical events stratified by the cutoff of S-wave amplitude ((**A**) heart failure readmission; (**B**) percutaneous mitral valve repair; (**C**) cardiovascular death). Odds ratio of S > 1.3 mV for heart failure readmission was 0.31 [0.10–0.95], *p* = 0.040, and for cardiovascular death was 0.11 [0.01–0.95], *p* = 0.045.

Cumulative incidences of cardiovascular death, heart failure readmission, and PMVR were evaluated as secondary endpoints. S-wave amplitude in right precordial leads >1.3 mV was significantly associated with lower incidence of heart failure readmission and PMVR during the five-year follow-up (*p* = 0.032 and *p* = 0.011, respectively, Figure 3A,B) but not of cardiovascular death (*p* = 0.112, Figure 3C). Of note, among 12 patients with grade 3–4 MR, all 6 patients with high S-wave amplitude could avoid PMVR.

## 3. Discussion

The present study demonstrates the association between baseline S-wave amplitude in right precordial leads and MR reduction following CRT. Although there was no significant difference in baseline left ventricular function between those with and without MR reduction, the higher S-wave amplitude in right precordial leads was associated with MR reduction independently as well as favorable clinical outcomes following CRT implantation.

### 3.1. Implication of S-Wave Amplitude in Right Precordial Leads for Functional MR

Intraventricular dyssynchrony in the left ventricle generates an incomplete mitral valve closure, leading to functional MR [9]. In particular, wide QRS duration in patients with LBBB or right ventricular pacing are associated with functional MR severity [10]. Good response to CRT can reduce functional MR by ameliorating left ventricular dyssynchrony [1,3,11]. Therefore, predictors of CRT response might have a considerable association with MR reduction following CRT implantation.

Wide QRS duration, LBBB, and left axis deviation are well-known predictors of CRT response, whereas this study failed to demonstrate their significant impact [12,13,14]. Left atrial volume has been documented as being a predictor of CRT responders, but left atrial diameter in this study was not associated with MR reduction [15]. These parameters are already established indications of CRT implantation, and most of our patients already satisfied them.

Instead, only the S-wave amplitude in right precordial leads was a robust independent predictor of MR reduction. In general, the S-wave in right precordial leads is affected by the left ventricular electrical delay, typically in LBBB [16]. Some of the patients with LBBB or right ventricular pacing dependence did not achieve MR reduction, meaning that S-wave amplitude might have an additive predictive power in addition to the morphological typing. We speculated that S-wave amplitude in right precordial leads might be affected by the severity of conduction disturbance in the left ventricle, irrespective of the type of bundle branch block or right ventricular pacing [17]. The severity of conduction disturbance has been considered as one of the most important parameters of CRT response as well as the mechanisms of functional MR [9]. Notably, this study demonstrated that the left ventricular reverse remodeling indices were not different between with and without MR reduction. As observed in another study, the papillary muscle dyssynchrony, which would be indicated by the high S-wave amplitude, might be independent of left ventricular remodeling [17].

### 3.2. The Proposed Strategy

**Figure 4:**
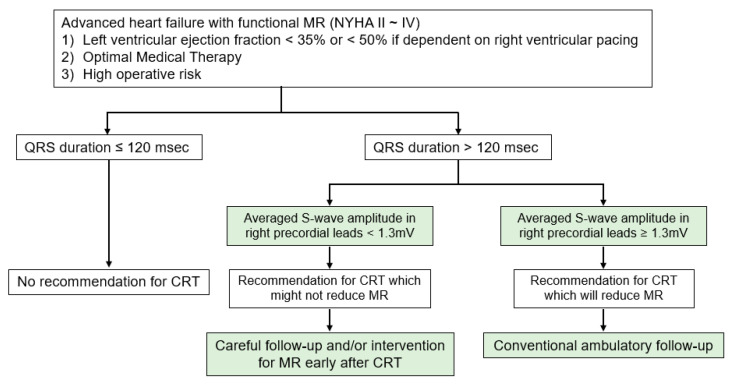
The proposed management strategy of functional mitral regurgitation in patients with advanced heart failure. CRT = cardiac resynchronization therapy and MR = mitral regurgitation.

We propose a simple practical strategy (Figure 4). If patients have LVEF <35% (or <50% if dependent on right ventricular pacing), QRS duration is the first checkpoint. In the presence of wide QRS duration, current guidelines suggest CRT implantation irrespective of MR (conventional recommendation) [4,5,6]. Furthermore, we propose an additional ECG marker, S-wave amplitude in right precordial leads, that further discriminates optimal patients who can enjoy the MR reduction as well as favorable clinical outcomes following CRT implantation (novel recommendation). 

We hypothesize that the novel marker might be a useful tool for the discrimination of PMVR along with CRT from CRT alone. For those without high S-wave, concomitant or early intervention to MR following CRT implantation is highly encouraged to prevent hemodynamic deterioration due to persistent or worsening MR in the near future.

### 3.3. Study Limitations

The present study has several limitations. First, this was a single-center retrospective observational study consisting of a small sample size, and there may be a selection bias. Second, S-wave amplitude might be affected by several factors, such as pericardial effusion, obesity, and pulmonary emphysema. Third, larger studies for detecting differences in the predictive power of MR reduction between S-wave amplitude in right precordial leads versus LBBB and right ventricular pacing dependence are also required. A QRS morphology sub-analysis in larger cohorts would give us more robust evidence. Fourth, since half of the subjects’ severity of MR was grade 1 in the present study, we could not discuss the indication of PMVR versus CRT. Further prospective multicenter registries, predominantly including subjects with severe MR for which PMVR is indicated, are needed. Finally, no information on left ventricular lead positions or gene mutations that might affect ventricular reverse remodeling and MR reduction was evaluated.

## 4. Methods

### 4.1. Study Population

Consecutive patients with advanced heart failure and functional MR who received CRT implantation between March 2010 and March 2021 at our institute were included retrospectively. All patients met the following criteria at baseline: (1) New York Heart Association functional class II-IV; (2) left ventricular ejection fraction (LVEF) ≤35% or <50% if dependent on right ventricular apical pacing rhythm; (3) QRS duration ≥120 ms; and (4) equal or greater than mild functional MR. The present study was approved by the institutional review board at the University of Toyama. Informed consent was obtained from all patients. 

### 4.2. Implant Procedure

Adequately trained board-certified cardiologists implanted the devices in the enrolled subjects. Any commercially available leads were implanted with a trans-subclavian venous approach. All the enrolled patients were successfully implanted with left ventricular leads under the fluoroscopy guide at the angles of left anterior oblique 45° and right anterior oblique 35° to document lead direction. Clinicians decided on CRT optimization methods chosen from QRS narrowing, trans-thoracic echo guiding, or automatic algorithm recommended by each manufacturer.

### 4.3. Baseline Clinical Characteristics

Baseline clinical characteristics, including demographics and laboratory data, were retrieved from the electronic medical record.

### 4.4. Standard 12-Lead Electrocardiograms

Variables such as PR interval except for atrial fibrillation, QRS axis, QRS duration in lead II, QRS morphology classified into right bundle brunch block, left bundle brunch block (LBBB), and intraventricular conduction disturbance were measured. In precordial leads, S-wave amplitude in leads V1 to V3 were measured and averaged according to the previous paper [18].

### 4.5. Echocardiograms and Assessment of MR

Echocardiographic parameters such as left atrial dimension, left ventricular end-diastolic dimension, left ventricular end-systolic dimension, left ventricular end-systolic volume, LVEF, and the severity of MR, which was evaluated using the regurgitant jet area for qualitative assessment and proximal isovelocity surface area for quantitating assessment according to the consensus guidelines, were collected at baseline and six months later [19,20]. MR severity was classified in the following five groups: none = 0, mild = 1, moderate = 2, moderate to severe = 3, and severe = 4. S.T. and N.F., who were blind to electrocardiographic parameters, judged the final comprehensive evaluation using echocardiographic parameters and clinical findings.

### 4.6. Clinical Outcomes

A reduction in MR ≥ 1 grade after six months, compared with baseline, was defined as a primary endpoint [21]. Simultaneously, biventricular pacing rates obtained by CRT recordings were also evaluated. Clinical events including cardiovascular death, worsening of heart failure requiring unplanned hospitalization, and PMVR using the MitraClip device within five-year follow-up were counted as secondary endpoints. Heart transplantation and left ventricular assist device implantation were included in the event of cardiovascular death.

### 4.7. Statistical Analysis

Two-sided *p*-value <0.05 was considered statistically significant. Data analysis was performed using JMP ver. 13.0.0 (SAS, Cary, NC). Data were expressed as the mean and standard deviation for normally distributed variables and as the median and interquartile range for non-normally distributed data. Continuous data were compared using t-test or the Mann–Whitney’s test, as appropriate. Categorical data were expressed as numbers and percentages and compared using Chi-squared test. 

Logistic regression analyses were performed to investigate the impact of baseline variables, including S-wave amplitude, on the reduction in MR grade following CRT implantation. Univariate analyses were performed for those with *p* < 0.05 in the comparison study. Since they were considered as the important parameters affecting the response to CRT, baseline rhythm, right bundle branch block (RBBB), and left axis deviation of QRS axis were also included in the logistic regression analysis. Multivariate analysis was performed for those with *p* value < 0.05 in the univariate analyses, including S-wave amplitude. As for QRS morphology, LBBB, which is an established predictor of CRT response, was chosen as a parameter for multivariate analysis, irrespective of significance. Receiver operating characteristics analysis was performed to calculate a cutoff of S-wave amplitude to predict post-CRT MR improvement. Cumulative incidence of clinical events was stratified by the cutoff of S-wave amplitude and compared between the two groups using a log-rank test.

## 5. Conclusions

The high S-wave amplitude in right precordial leads would play a key role in identifying patients who would benefit from MR reduction and favorable clinical outcomes following CRT implantation alone without concomitant PMVR.

## Figures and Tables

**Table 1:** Comparison of characteristics in patients with and without MR reduction.

Variable	Overall (*N* = 35)	With MR Reduction (*N* = 17)	Without MR Reduction (*N* = 18)	*p*-Value
*Demographics*				
Age, years	71 [65–78]	73 [67–81]	70 [60–76]	0.306
Male (%)	18 (51)	6 (35)	12 (67)	0.063
Body mass index, kg/m^2^	20.7 ± 3.7	20.0 ± 3.5	21.5 ± 3.7	0.239
Ischemic etiology (%)	4 (11)	3 (18)	1 (6)	0.261
Persistent atrial fibrillation/flutter (%)	7 (20)	3 (18)	4 (22)	0.735
CRT-P (%)	7 (20)	5 (29)	2 (11)	0.176
ICD for primary prevention (%)	17 (61)	9 (75)	8 (50)	0.180
*Comorbidity*				
Chronic kidney disease (%)	11 (31)	4 (24)	7 (39)	0.328
Diabetes mellitus (%)	6 (17)	4 (24)	2 (11)	0.330
NYHA functional classification IV (%)	4 (11)	2 (12)	2 (11)	0.952
Pre-implantation vital signs				
Heart rate, bpm	73 ± 15	77 ± 17	69 ± 13	0.145
Systolic blood pressure, mmHg	107 ± 19	105 ± 19	108 ± 18	0.642
Diastolic blood pressure, mmHg	66 ± 12	62 ± 11	70 ± 12	0.056
*Medications*				
ACE-I or ARB (%)	31 (89)	15 (88)	16 (89)	0.952
Beta-blockers (%)	27 (77)	14 (82)	13 (72)	0.476
Loops (%)	29 (83)	15 (88)	14 (78)	0.412
Digitalis (%)	2 (6)	1 (6)	1 (6)	0.967
Inotropes (%)	5 (14)	2 (12)	3 (17)	0.679
Amiodarone (%)	11 (31)	4 (24)	7 (39)	0.328
*Laboratory data*				
Serum albumin, g/dL	3.7 ± 0.4	3.7 ± 0.4	3.7 ± 0.4	0.840
Serum total bilirubin, mg/dL	0.6 [0.5–0.9]	0.6 [0.4–0.8]	0.6 [0.5–1.0]	0.414
Serum creatinine, mg/dL	0.9 [0.7–1.3]	0.8 [0.7–1.2]	1.1 [0.8–1.5]	0.129
Estimated GFR, mL/min/1.73 m^2^	50.4 ± 21.4	55.8 ± 17.5	45.9 ± 24.0	0.250
Serum sodium, mEq/L	138 [134–140]	138 [134–140]	139 [136–140]	0.405
Hemoglobin, g/dL	12.5 ± 2.0	11.9 ± 1.8	13.2 ± 2.0	0.060
Plasma B-type natriuretic peptide, pg/mL	482 [167–909]	488 [171–944]	384 [162–950]	0.918
*Echocardiographic parameters*				
Left atrial dimension, mm	47 ± 9	43 ± 8	50 ± 9	0.031
Left ventricular end-diastolic dimension, mm	63 [56–68]	62 [57–73]	64 [56–66]	0.791
Left ventricular end-systolic dimension, mm	52 [48–61]	53 [49–63]	52 [47–58]	0.488
Left ventricular end-systolic volume, mL	132 [106–189]	144 [110–199]	130 [101–168]	0.427
Left ventricular ejection fraction, %	27 ± 8	25 ± 7	29 ± 9	0.164
Degree of MR				0.689
Grade 1	19 (54)	10 (59)	9 (50)	
Grade 2	4 (11)	1 (6)	3 (17)	
Grade 3	3 (9)	2 (12)	1 (6)	
Grade 4	9 (26)	4 (24)	5 (28)	
*Electrocardiographic parameters*				
QRS morphology (%)				0.001
Left bundle branch block (%)	10 (29)	9 (53)	1 (6)	0.002
Right bundle branch block (%)	8 (23)	1 (6)	7 (39)	0.020
Intraventricular conduction disturbance (%)	5 (14)	0 (0)	5 (28)	0.019
Right ventricular pacing (%)	12 (34)	7 (41)	5 (28)	0.404
Axis, degree	0 [–63–0]	0 [−29–14]	−32 [−78–0]	0.052
PR interval, msec	208 [185–287]	200 [182–247]	250 [186–311]	0.157
QRS duration in II, msec	174 ± 29	174 ± 31	174 ± 27	0.976
Averaged S-wave amplitude in V1–3, mV	1.3 [0.7–3.0]	3.0 [1.5–3.5]	0.7 [0.4–1.2]	<0.001
Biventricular pacing rate at six months, %	99.0 [98.2–99.8]	99.0 [99.0–100]	99.0 [96.5–99.3]	0.095

ACE = angiotensin-converting enzyme; ARB = angiotensin receptor blocker; CRT = cardiac resynchronization therapy, CRT-P = cardiac resynchronization therapy pacemaker; ICD = implantable cardioverter defibrillator; MR = mitral regurgitation; NYHA = New York Heart Association; GFR = glomerular filtration rate.

**Table 2:** Logistic regression analyses of a reduction in MR.

Variables	Univariable		Multivariable	
	OR [95%CI]	*p*-Value	OR [95%CI]	*p*-Value
Atrial fibrillation/flutter	0.75 [0.14–3.98]	0.736		
Left atrial diameter	0.91 [0.83–1.00]	0.046	0.94 [0.81–1.09]	0.376
Left bundle branch block	19.1 [2.06–177.92]	0.010	7.24 [0.40–131.71]	0.181
Right bundle branch block	0.08 [0.01–0.72]	0.025	0.36 [0.02–5.88]	0.477
Right ventricular pacing	1.82 [0.44–7.48]	0.406		
QRS axis > −30 degrees	0.31 [0.07–1.31]	0.112		
Averaged S-wave amplitude in V1–3	6.52 [1.88–22.62]	0.003	14.00 [1.65–119.00]	0.016

CI = confidence interval; OR = odds ratio.

## Data Availability

The data presented in this study are available on request from the corresponding author.

## Abbreviations

CRT	cardiac resynchronization therapy
ECG	electrocardiogram
LBBB	left bundle branch block
LVEF	left ventricular ejection fraction
MR	mitral regurgitation
PMVR	percutaneous mitral valve repair
RBBB	right bundle branch block
